# Modeling Oxidation of AlCoCrFeNi High-Entropy Alloy Using Stochastic Cellular Automata

**DOI:** 10.3390/e24091263

**Published:** 2022-09-08

**Authors:** Indranil Roy, Pratik K. Ray, Ganesh Balasubramanian

**Affiliations:** 1 Department of Mechanical Engineering, Lehigh University, 19 Memorial Drive W, Bethlehem, PA 18015, USA; 2 Department of Metallurgical and Materials Engineering, Indian Institute of Technology—Ropar, Rupnagar 140001, Punjab, India

**Keywords:** oxidation, high-entropy alloy, mesoscale, cellular automata

## Abstract

Together with the thermodynamics and kinetics, the complex microstructure of high-entropy alloys (HEAs) exerts a significant influence on the associated oxidation mechanisms in these concentrated solid solutions. To describe the surface oxidation in AlCoCrFeNi HEA, we employed a stochastic cellular automata model that replicates the mesoscale structures that form. The model benefits from diffusion coefficients of the principal elements through the native oxides predicted by using molecular simulations. Through our examination of the oxidation behavior as a function of the alloy composition, we corroborated that the oxide scale growth is a function of the complex chemistry and resultant microstructures. The effect of heat treatment on these alloys is also simulated by using reconstructed experimental micrographs. When they are in a single-crystal structure, no segregation is noted for α-Al_2_O_3_ and Cr_2_O_3_, which are the primary scale-forming oxides. However, a coexistent separation between Al_2_O_3_ and Cr_2_O_3_ oxide scales with the Al-Ni- and Cr-Fe-rich regions is predicted when phase-separated microstructures are incorporated into the model.

## 1. Introduction

Alloys used in extreme environments, such as turbines and aero-engines, require excellent high-temperature strength and oxidation resistance. Recent progress in high-entropy alloys (HEAs), which have five or more elements present in near-equimolar proportions, has resulted in several alloys with excellent high-temperature strength. However, the oxidation and corrosion behavior of HEAs is still poorly understood and modeled. Oxidation is an extremely complex process where kinetics, thermodynamics, and microstructure play a competing role in forming the passivating oxide layer, which prevents a catastrophic oxidation [1,2]. Challenges in modeling the oxidation behavior of HEAs are numerous, including understanding the role of chemistry, microstructure, grain boundaries, associated volume changes, stresses in the oxide scale, and the environment itself, along with the thermodynamic stabilities at elevated temperature and the kinetics of the process. We attempt a piecewise approach toward modeling the oxidation behavior of HEAs and to address a critical question in this work—*how do the composition and the microstructure affect the oxidation behavior of the HEAs?* Forming an ensemble model that will capture the effect of all of these factors and generate insights that are directly comparable to experimental results is necessary for understanding the oxidation of HEAs [3].

The individual factors mentioned above have been well-assessed experimentally for conventional alloys. For instance, Ray et al. demonstrated issues with the dendritic microstructures of NiAl-Mo alloys [4]. The role of the microstructural length scales has also been investigated experimentally in Mo-Si-B alloys by various researchers [5,6], including one of the authors of this paper. Typically, passivating elements in traditional high-temperature materials are chosen from Al, Cr, or Si, which form α-Al_2_O_3_, Cr_2_O_3_, and SiO_2,_ respectively. Several HEAs contain Al and Cr as principal components. Hence, here we focus on a particular system (AlCoCrFeNi) that oxidizes to form a mixed oxide scale with α-Al_2_O_3_ and Cr_2_O_3_ as the major constituents. Park et al. demonstrated the increase in oxidation resistance with the addition of Al- in Ni-based superalloys [7]. Similar conclusions are drawn by Saber et al. [8] for the addition of Cr into IN617. A consistent effort has been put into improving the oxidation resistance of Ni-based superalloys by adding Al and Cr in the past [9,10]. Fe-based alloys are well studied in terms of oxidation resistance, with FeCrAl being one of the top candidates for both high- and low-temperature oxidation-resistant material [11,12]. At high temperatures (>1000 °C), a thin α-Al_2_O_3_ acts as a diffusion barrier between oxide and bulk metal, protecting the oxidation. In low temperatures (<500 °C), Cr_2_O_3_ creates a similar permission barrier that prevents the oxidation of FeCrAl alloys [13]. Like Ni- and Fe-based alloys, HEAs have demonstrated superior mechanical properties; hence, they became a strong candidate as a structural material, starting in the early 2000s [14,15]. The oxidation of Cr- and Al-based alloys is particularly interesting due to their good oxidation-resistance capabilities [16]. Recently, there have been more studies on the oxidation of the AlCoCrFeNi system [17,18,19]. As the composition space increases with the introduction of new HEAs, it becomes increasingly challenging to experimentally test them in variable oxidizing environments.

Modeling oxidation can be an intelligent and efficient way to predict the environmental resistance of the alloys and screen candidate materials for high-temperature applications. Both mathematical models with numerical implementation and computational material modeling have been performed in the past, either to simulate or gain more insight into the alloy oxidation [20,21]. Wagner’s theory is generally efficient in explaining internal oxidation mathematically for binary alloys [22,23]. In recent years, attempts have been made to extend Wagner’s internal-oxidation model for the external oxidation [24], but the efforts are very limited to the phenomenon of diffusion and reaction. In computational material space, the oxidation modeling of alloys is traditionally performed by using thermodynamic analysis, first-principles energetic calculations, atomistic modeling [25,26], and continuum-level modeling. A large part of the computational efforts on modeling alloy oxidation has been restricted to binary alloys, although recent efforts have focused on ternary systems [21,27], and even HEAs, as well [28,29]. In a nutshell, CALPHAD-based modeling and ab initio modeling on the oxidation of alloys provide insight into the reaction and thermodynamic behaviors of oxides and their interaction with the unoxidized alloy [30]. On the other hand, atomistic modeling, such as molecular dynamics, can help explain the kinetics, e.g., the diffusion and affinity of different atoms involved in the reaction process [31].

Although the existing oxidation modeling approaches can explain the fundamentals of oxidation, two major gaps need to be addressed. First, the existing models are focused on a very specific understanding of either thermodynamics or kinetics of oxidation. For example, thermodynamic calculation on oxides can help us identify the stable oxides and the oxygen position on the surface for the reaction to occur [32]. Still, it does not provide information on how the oxide will grow. The second limitation of existing models is the inability to capture the microstructural features. It has been established in the past that the microstructure has important implication information of oxides in any alloy [4,5,6,33,34,35]. Therefore, accounting for microstructural inhomogeneity is especially important, along with chemistry to model oxidation.

Here, we present a mesoscale modeling approach based on stochastic Cellular Automata (CA) to describe the oxidation mechanism in the AlCoCrFeNi HEA by accounting for the thermodynamics, kinetics, and microstructural features associated with oxidation. AlCoCrFeNi oxidation is an experimentally well-studied system, allowing our model to be compared against the available data. We first investigate the effects of different alloy chemistries, followed by examining the effect of different multi-phase microstructures.

## 2. Computational Method

Stochastic CA is used for modeling the oxidation of compositionally complex alloys, as it can reproduce both the thermodynamics and kinetics of oxidation, together with the relevant microstructures [36]. Being a mesoscale model, CA provides flexibility to import data from lower-length-scale models, which are essential for capturing the effect of oxidation in full. We varied the size of the simulation cell from 50 × 50 to 200 × 200. It was found that the 100 × 50 matrix size is spatially large enough to capture the oxidation processes of interest, in general, and adding more cells only increases computational time, with no next effect on simulated results. Wherever we have used experimental micrographs to model the process, we have modified the simulation cell size to match the aspect ratios of the micrographs. The model adopted in this work was built closely upon our previous efforts of modeling the oxidation behavior of NiAl and Hf-doped NiAl and is explained in detail elsewhere [36] (Figure 2 of Reference [33] shows a clear flowchart of the model). In this study, we extended the same model for a five-element alloy system. The oxidation reaction with pure metal is assumed to be instantaneous and can only occur in the Von Neumann neighborhood. Apart from oxidation, the reduction of Fe, Co, and Ni oxides is considered in the presence of Al and Cr. For AlCoCrFeNi HEA, spinels typically form at a low partial pressure of oxygen during the initial oxidation state [37], and the growth of the oxide scale is not determined by them [38,39]. Therefore, the formation of spinels is ignored in this particular work. The downward diffusion of oxygen and upward diffusion of metals are added as a probability, and the diffusion coefficient values are adopted from Reference [31]. Table 1 shows the diffusion coefficients and the corresponding transformation into probability values in the CA model.

**Figure 1:**
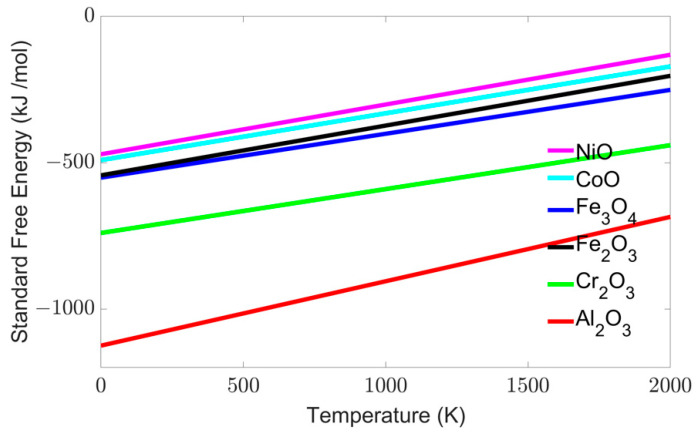
Ellingham diagram of native oxides for the principal elements in AlCoCrFeNi HEA [40]. The lower free energy in the Ellingham diagram signifies an enhanced thermodynamic stability.

The output of the models is provided in three primary forms, (a) qualitative microstructure and quantitative (b) thickness, and (c) concentration variation as a function of time. The microstructures need no description, as they are provided in a clearly color-coded format. Oxides are denoted in shades of red, while the metal is denoted by using different shades of blue. The color intensity describes the stability of oxides in the following order: Al > Cr > Fe > Co > Ni oxides [40], as shown in Figure 1.

The metals are also signified by the same approach, i.e., dark to light blue: Al > Cr > Fe > Co > Ni. The air is yellow and green. The average thickness is calculated by counting the thickness of the oxide in each column and dividing the resulting value by the total number of columns. The concentration of individual oxides is calculated by using the following formula:

(1) $$C_{A\;oxide}=\frac{Total\;Cells\;occupied\;by\;A\;oxide}{\sum_{X=Al,\;Cr,Fe,Co,Ni}Total\;Cells\;occupied\;by\;X\;oxide}$$

The spatial and temporal scaling was adopted from the model created for Ni-Al binary alloy previously [36].

## 3. Results and Discussion

We attempted to gain insights into the oxidation behavior of AlCoCrFeNi alloys by investigating the role of alloy chemistry and microstructure. In order to understand the role of alloy chemistry, we focused on a “single-crystal” version of the alloy; we varied the chemistry, without creating any variations in the microstructure, in an effort to isolate the chemical effects computationally. The compositional variations are primarily investigated through (a) variation in the Al:Cr ratio and (b) variation in the Fe:Co:Ni ratio. The microstructure effects are studied by utilizing experimental micrographs for creating the simulation cell. Primarily, two different sets of microstructures are considered. Typically, as-cast AlCoCrFeNi alloys crystallize with a dendritic microstructure, with distinct chemistries in the dendritic core and the inter-dendritic regions [41]. On the other hand, rapidly cooled and heat-treated/aged alloys can exhibit a precipitate/matrix microstructure [42]. In this work, both microstructures were investigated computationally to understand the oxidation behavior of the alloy.

### 3.1. Oxidation of the Equiatomic AlCoCrFeNi Alloy

**Figure 2:**
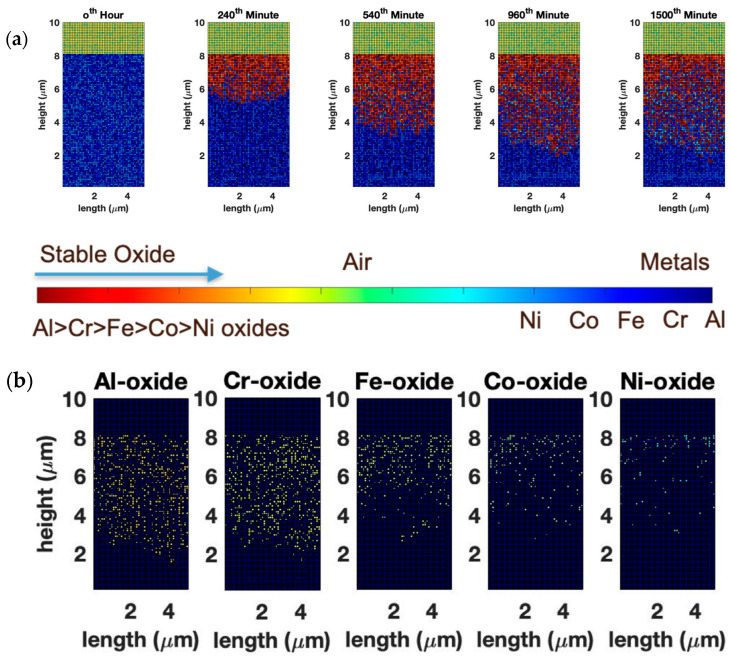
(**a**) Temporal evolution of Al_x_Cr_(40__−x)_Fe_20_Co_20_Ni_20_ alloy (where x = 20) oxide scale obtained from CA modeling. (**b**) Micrographs show the elemental distribution of different oxides in the scale. The slow diffusivities of the metal cations render the composition of the top layer to be relatively unchanged, but deeper into the oxide scale, Al and Cr emerge as the favored oxides, with only a marginal presence of Fe, Co, and Ni oxides.

**Figure 3:**
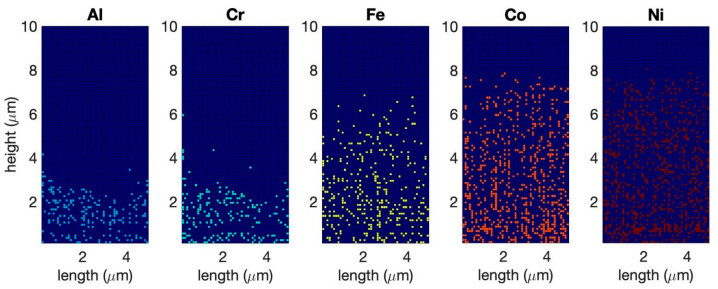
Distribution of unoxidized metals after 25 h of oxidation of equimolar AlCoCrFeNi HEA at 1000 K. There is a significant depletion of the primary scale-forming elements, Al and Cr, while a significant amount of unoxidized Fe, Co, and Ni remains distributed throughout the subscale region.

We first built the CA model of equimolar AlCoCrFeNi HEA wherein the species were randomly distributed across the simulation domain with uniform probability. As seen from the compositional distribution of the oxide, Al and Cr oxides are the primary component of the scale, which agrees with the experimental literature [37]. We do not notice significant segregation of α-Al_2_O_3_ and Cr_2_O_3_ in the oxide scale after 25 h of oxidation at 1000 K (Figure 2a). During the initial stages of oxidation, the top layer of the alloy oxidizes upon contact with the atmospheric oxygen. At this stage, the oxidation behavior is least influenced by the thermodynamic effects. In Figure 2b, this becomes fairly evident, as we compare the spatial distribution of different oxides. In the top-most layer, we have a relatively even distribution of all the oxides. However, with the progress of oxidation, both kinetics (diffusion of anionic and cationic species through the oxide scale) and thermodynamics (stability of the oxides, incorporating the probability of the reduction of previously formed oxides by more stable oxides) affect the structure of the oxide scale. Due to the relatively slow diffusivities of the metal cations, the top layer of cells remains relatively unchanged, but as we go deeper into the oxide scale, it becomes evident that Al and Cr are the favored oxides, with progressively less Fe_2_O_3_, CoO_2_, and NiO forming. Hypothetically, if a chemical analysis of such an oxide scale is performed, one would see a greater amount of oxides other than α-Al_2_O_3_ and Cr_2_O_3_ by using near-surface chemical analysis techniques such as X-Ray Photoelectron Spectroscopy (XPS), as opposed to techniques such as Energy- Dispersive X-Ray Spectroscopy (EDS), where the analysis volume is larger, in which case α-Al_2_O_3_ and Cr_2_O_3_ would dominate. Garg et al. [43] experimentally found a considerable amount of Ni, Co, and Fe oxides in XPS, along with Cr and Al oxides, thus corroborating our findings that Ni, Co, and Fe oxides will be present near the surface. EDS studies from the surface, on the other hand, show a greater amount of Al and Cr in the oxide scale. Figure 3 shows a complementary elemental distribution of the unoxidized alloy. It can be seen clearly that there is a significant depletion of the Al and Cr, which are the primary scale-forming elements, with a marginally greater depletion of Al with respect to Cr. A depletion of Fe, Co, and Ni is seen in the near-surface region where the scale has formed in the initial stages of oxidation stochastically. However, as we go deeper into the alloy, a significant amount of unoxidized Fe, Co, and Ni can be seen distributed throughout, especially in the subscale region.

**Figure 4:**
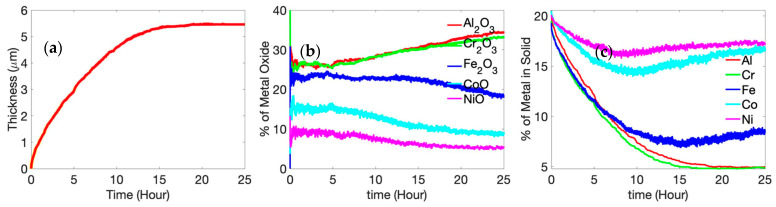
(**a**) Time-dependent average thickness, (**b**) metal oxide concentration, and (**c**) unoxidized metal concentration variation of Al_x_Cr_(40__−x)_ Fe_20_Co_20_Ni_20_ alloy (where x = 20) at 1000 K. The oxidation kinetics assume a parabolic profile since the single-crystal motif is considered in the simulations where the only diffusion mechanism is bulk (lattice) diffusion, with grain boundaries (and, hence, grain-boundary diffusion) being absent.

The kinetics of the oxidation process is depicted in Figure 4, where we show the temporal variation of the oxide-scale thickness, oxide-scale composition, and unoxidized alloy composition. The thickness of the oxide scale increases in a parabolic manner, as reported in previous experiments [37,38,39,44]. Parabolic oxidation kinetics are fairly typical in diffusion-controlled processes. This effect is perhaps further accentuated in these simulations since we chose to consider a “single-crystal” motif, where the only diffusion mechanism is bulk (lattice) diffusion with grain boundaries (and, hence, grain-boundary diffusion) being absent in a single-crystal simulation cell. After 25 h of oxidation, the oxide layer primarily consists of α-Al_2_O_3_ and Cr_2_O_3_. Previous experimental reports on high-temperature oxidation of AlCoCrFeNi HEA indicate the same [37,38,45]. Butler et al. [37] reported an external chromia layer, followed by an internal Alumina, after 50 h of oxidation of equimolar AlCoCrFeNi alloy at 1323 K (1050 °C). Hall et al. [44] provided a detailed explanation for protective α-Al_2_O_3_ formation primarily through the grain-boundary diffusion of Al (a mechanism not considered in a single-crystal model); this perhaps explains why we observed a greater extent of Cr_2_O_3_ in the oxide scale in our simulations. At the initial stage of oxidation (till 1 h), experimental evidence suggests that all of the oxides form [44], and this is in agreement with our simulation results (Figure 4). It is seen that, among the oxides of Fe, Co, and Ni, a significant amount of iron oxide is present until about 5 h of oxidation. After 5 h, enough Fe oxide has formed to dissociate into Fe, thus resulting in an increase of the Al and Cr oxide forming through a reduction reaction [44]. Ni and Co oxide concentrations are 20% and 15%, respectively, for the first 5 h of oxidation. After 5 h, and with an increase in time, Ni, Co, and Fe oxide concentrations decrease as they become reduced with increasing amounts of Al and Cr oxides forming in their place.

In our simulations, we did not observe the segregation of alumina and chromia in the oxide layer. We believe that the absence of grain boundary reduces faster upward Al transport, limiting it only through the lattice. Therefore, a mixed oxide layer of α-Al_2_O_3_ and Cr_2_O_3_ was found with no clear segregation. Moreover, a majority of the studies that report clear segregation of α-Al_2_O_3_ and Cr_2_O_3_ are performed above 1323 K. At 1000 K, there is not enough kinetic activity; that is, high diffusion of Al through the HEA (Table 2) is occurring, and this will segregate the layers. By tracking the presence of metal, we observed the entrapment of Ni and Co into the oxide scale (Figure 4b), as reported by Hall et al. [44]. The illustration also shows the depletion of Al and Cr below the oxide scale.

#### 3.1.1. Effect of the Al:Cr Ratio

**Figure 5:**
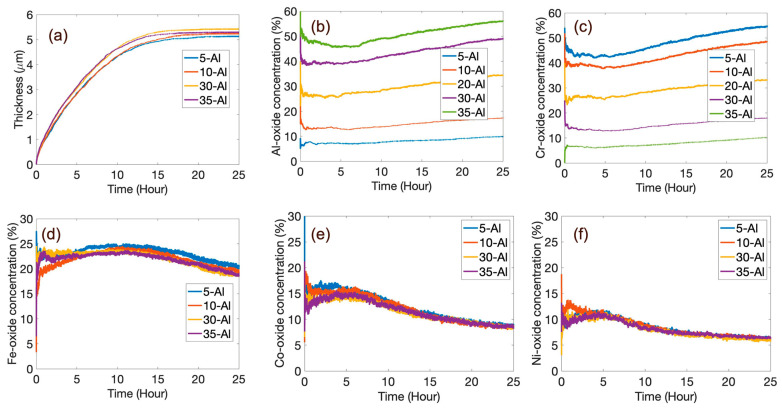
Comparison of (**a**) oxide thickness and (**b**–**f**) individual elemental oxide (Al-Cr-Fe-Co-Ni, respectively) concentration with time for Al_x_Cr_(40__−x)_ Fe_20_Co_20_Ni_20_, where x = 5, 10, 30, and 35. Equimolar alloy oxide concentration is also shown in the case of (**b**) Al and (**c**) Cr oxide for comparison. Since there are no variations in Fe, Co, and Ni fractions with variation in x, the corresponding oxide concentrations (**b**–**f**) remain unaltered with changes in Al:Cr ratios.

We performed further simulations by varying the Al:Cr ratio, while keeping Fe, Co, and Ni at 20 at.% each. For Al_x_Cr_(40__−x)_ Fe_20_Co_20_Ni_20_, x is varied as 5, 10, 30, and 35 to capture the effect of the Al:Cr ratio. The results of these simulations are summarized in Figure 5. A very small variation (±2.85% with respect to equimolar HEA) in oxide-scale thickness is observed after 25 h at 1000 K. For the high-Al-containing alloys, the oxide kinetics has reached a steady state (as the thickness plateaus) a little faster than for the low-Al-containing alloys. The fast-forming α-Al_2_O_3_ layers in the high-Al-containing alloys help form passivating oxide scales quicker than in the low-Al-containing ones. We also plotted the time-dependent individual oxide concentration for all the different alloy chemistries. Figure 5b,c show the concentration of alumina and chromia in the oxide scale as the Al content is changed. From these simulations, it appears that changing the Al:Cr ratio does not affect the total amount of the more passivating oxides (i.e., the α-Al_2_O_3_ + Cr_2_O_3_ amount). The trends seen in Figure 5b,c are virtually identical. A subtle difference can be observed through the fact that the amount of alumina for a given Al content is marginally higher than the amount of chromia for the same Cr content. This is likely a consequence of the greater thermodynamic stability of the alumina [12]. When sufficient quantities of different metals are present, the probabilities of formation for the various oxides are mapped corresponding to the respective stabilities of the individual oxides; hence, alumina, being more thermodynamically stable at high temperatures, forms in a relatively greater fraction than other oxides [46]. As there is no variation in Fe, Co, and Ni metal concentration, these oxide concentrations are unaltered with varying Al-to-Cr ratios and almost overlap each other (Figure 5d–f). A careful analysis of the general trend of the time-dependent concentration plot shows that, for the Al and Cr oxides, the concentration decreases slightly for the first couple of hours and then increases after 5 h. Previous experimental reports suggest the presence of thermodynamically unstable oxides (Figure 1), e.g., NiO at the initial stage of oxidation that gets dissolved as time progresses [47]. Our model concurs with these findings, as the NiO and CoO concentration in the oxide scale increases for 5 h before decreasing monotonically. Fe oxide also follows a similar trend as NiO or CoO, but the inflection point is between 10 and 15 h, which is also likely a consequence of the higher thermodynamic stability of iron oxide as compared to cobalt or nickel oxides (hence, the slower rate of reduction of the iron oxides as compared to cobalt or nickel oxides). As a consequence of these dynamical changes in the initial stages of oxidation, the oxide-scale chemistry changes over time initially before stabilizing toward an Al- and Cr-rich scale chemistry. As reported by Listyawan et al. [48], our model also demonstrates a considerable presence of Fe_3_O_4_ and Fe_2_O_3_ in the oxide scale due to the high diffusion coefficient of Fe at low temperatures [31].

A careful observation of the time-dependent concentration change plots for varying Al to Cr ratios shows an interesting difference between Al and Cr oxides with the rest of the oxides. The fluctuation of the concentration of thermodynamically unstable oxide (Fe, Co, and Ni oxides) is higher than thermodynamically stable oxides (Al and Cr oxides), making the lines of Figure 5d–f appear thicker than Figure 5b,c. Similar observations can be made for all the oxide concentration plots, as well. As Fe, Co, and Ni oxides are thermodynamically unstable, the formation and dissociation occur more frequently in these oxides as compared to Al and Cr oxides.

#### 3.1.2. Effect of Fe:Co:Ni

**Figure 6:**
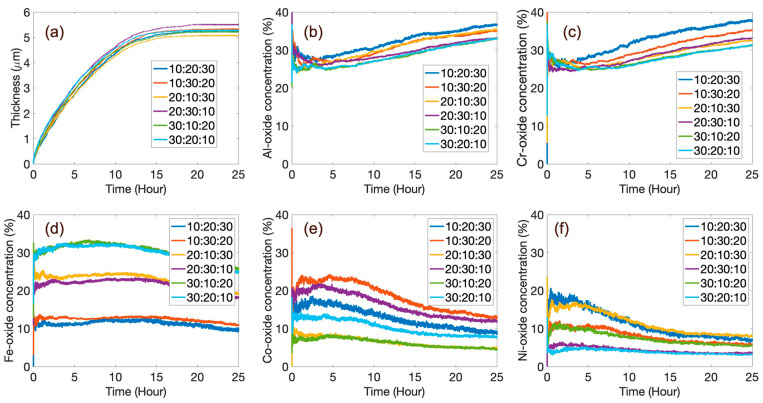
The kinetics of oxidation is shown for different Fe, Co, and Ni ratios, while keeping Cr and Al at 20 at.% each. The Fe:Co:Ni at.% values are described in the legend of the thickness (**a**) and oxide-concentration plots (**b**–**f**). Overall, the trends in the formation of alumina and chromia remain uniform with variations in the Fe:Co:Ni mass fractions in the alloy.

We performed a second set of simulations to study the effect of alloy chemistry. In the previous set of simulations, the Al:Cr ratio was varied, keeping the Fe, Co, and Ni content constant. Here, we kept the Al and Cr content constant at 20 at.% each and varied the Fe:Co:Ni ratio in all combinations of 10, 20, and 30 at.%. The chemical compositions are Al_20_Cr_20_Fe_10_Co_20_Ni_30_, Al_20_Cr_20_Fe_10_Co_30_Ni_20_, Al_20_Cr_20_Fe_20_Co_10_Ni_30_, Al_20_Cr_20_Fe_20_Co_30_Ni_10_, Al_20_Cr_20_Fe_30_Co_10_Ni_20_, and Al_20_Cr_20_Fe_30_Co_20_Ni_10_. No significant variation in oxide kinetics is observed for these changes (Figure 6). From Figure 6b,c, it becomes apparent that the concentration of alumina and chromia does not change significantly as the Fe:Co:Ni ratios are changed, although subtle changes can be observed. The lower the Fe content, the higher is the α-Al_2_O_3_ and Cr_2_O_3_ content in the alloy. This is consistent with the fact that, among the oxides of Fe, Co, and Ni, iron oxides (both Fe_3_O_4_ and Fe_2_O_3_) have the highest thermodynamic stability [30]. Therefore, iron oxides are reduced at a lower rate when they form. The concentration of the iron oxide is proportional to the concentration of Fe in the alloy. Hence, at a low Fe content, with a lower amount of iron oxide formed, greater amounts of CoO and NiO are formed, which are easier to reduce by Al and Cr (and, hence, the higher the concentrations of α-Al_2_O_3_ and Cr_2_O_3_ at lower Fe content). Overall, the trends of formation of α-Al_2_O_3_ and Cr_2_O_3_ remain more or less uniform with variations in the Fe:Co:Ni ratio. The oxides of Fe, Co, and Ni largely form according to their content in the alloy. When the Fe composition is kept at 30%, the iron oxide content seems identical regardless of the Co and Ni content, as seen in Figure 6d. The same can be observed for the other concentrations (Fe-10% and Fe-20%) studied here. Figure 6f indicates a similar variation of the NiO content, with the concentration of NiO being roughly identical for similar Ni content, regardless of Fe and Co content. The scatter, however, is somewhat larger than the case of iron oxide. This is not the case, however, for the CoO concentration, as seen in Figure 6e. While a larger Co content in the alloy results in a higher CoO concentration in the scale, the same values of Co content do not necessarily result in a similar CoO concentration according to the oxide scale. This is indeed puzzling, since the thermodynamic stability of CoO and NiO is nearly identical, with CoO having slightly higher stability. However, there is a significant difference in the thermodynamic stability of iron oxide and NiO. Hence, when the Fe content is higher for the same Co content, a smaller amount of CoO is formed. From these two sets of simulations, it becomes quite clear that tuning the Al:Cr content can significantly affect the structure of the oxide scale that forms. However, the effect of the Fe:Co:Ni ratio is relatively negligible (although Fe plays a greater role in affecting oxide-scale chemistry than Co and Ni). Similar results have been observed in FeCrAl-based alloys, where the oxide-scale structure is primarily biased through tuning the Al:Cr ratio [37].

### 3.2. Effect of Microstructure on the Oxidation Behavior

The single-crystal simulations provide useful insights into the effect of chemistry. Several HEAs (including the system under consideration), however, are multi-phase polycrystalline materials. The microstructure of such materials changes with changes in solidification conditions. While non-equilibrium cooling can result in extended solid solubilities and the formation of metastable solid solutions, the slower cooling rates more prevalent in casting operations result in vastly different microstructures. Often, the primary solidifying phase forms as dendrites, with the remaining liquid (with a markedly different composition from the dendrites) solidifying in the inert-dendritic regions. A number of researchers working on different alloy systems have in fact noted the effect of the alloy microstructure on the oxidation behavior [4,5,6]. Therefore, it is critical to have a model with the ability to capture the effect of such microstructural features. Two distinct microstructural features are addressed in two different length scales in this work. First, two differently heat-treated (1373 K and 1473 K for 3 h each) samples of AlCoCrFeNi equimolar alloy were considered for oxidation at 1000 K for 25 h. The choice of microstructures was predicated on the fact that these microstructures seem to be the most commonly observed for heat-treated samples. Heat-treated alloys, rather than as-cast alloys, were considered since rapidly quenched as-cast alloys have been reported to form a metastable single-phase BCC-like structure, which resembles the earlier simulations to a reasonable extent. It should be noted that heat treatment was not modeled by using CA, and we adopted only the digital micrographs from the experimental literature [41,42]. As a second step, we modeled the same in the dendrite core and interdendritic regions where the precipitate and matrix have varying concentrations. The previously discussed model was used in all the cases, except that the initial composition is not random and predefined according to the compositional distribution obtained from the Energy-Dispersive Spectroscopy (EDS) data reported in the experimental literature.

#### 3.2.1. Dendritic-Core and Interdendritic Microstructure

Studies on the AlCoCrFeNi alloy suggest two primary phases—an Al-Ni-rich dendritic phase and a Cr-Fe-rich interdendritic phase [41]. The digital microstructures of arc-melted and heat-treated samples are adopted from the literature of Munitz et al. [42].

**Figure 7:**
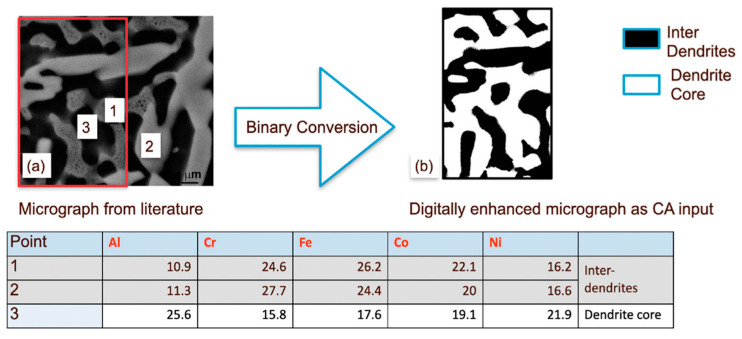
(**a**) Digital micrograph of AlCoCrFeNi alloy after heat treatment at 1373 °C for 3 h obtained from Reference [42] and converted into (**b**) a binary image, with compositions indicated in the table. The micrograph is cropped to a suitable dimension that can be used for the CA model. The model predictions do not show significant differences in oxidation kinetics compared to a single crystal.

**Figure 8:**
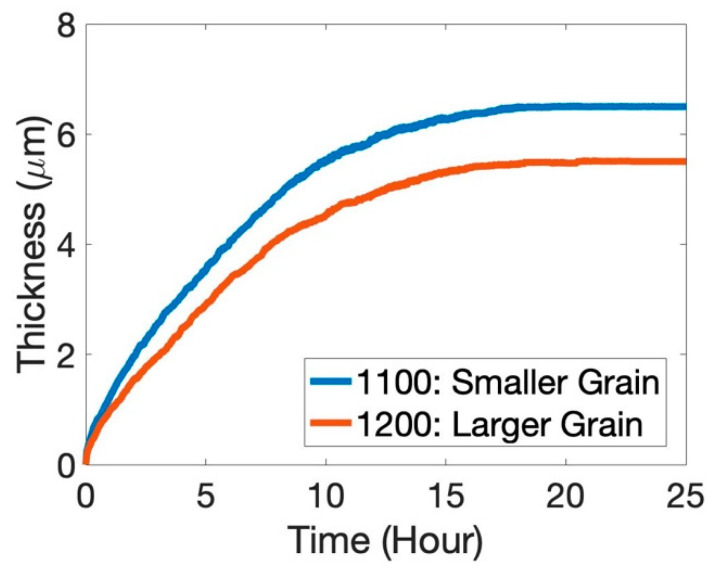
Comparison of oxide-scale thickness after 25 h of oxidation in air for 1100 °C (or 1373 K; blue) and 1200 °C (or 1473 K; red) heat-treated samples.

**Figure 9:**
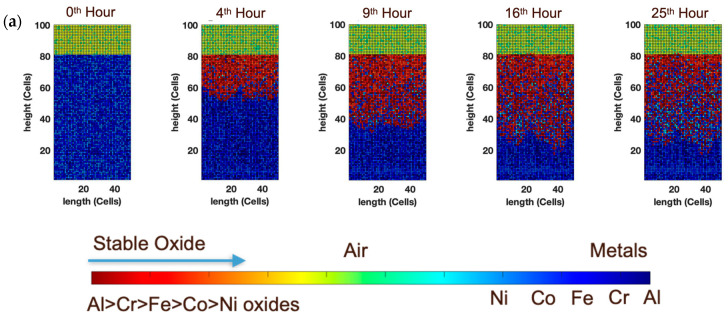

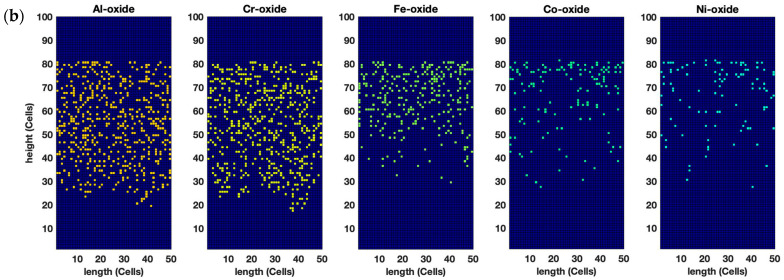
(**a**) Temporal evolution of AlCoCrFeNi alloy oxide scale obtained from CA modeling. (**b**) Micrographs show the elemental distribution of different oxides in the scale. With increasing grain size, the oxidation kinetics become sluggish due to reduced grain-boundary regions. Nevertheless, the oxide scale’s composition remains relatively invariant.

**Figure 10:**
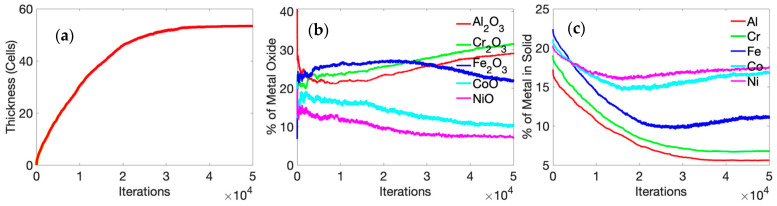
Time-dependent (**a**) average thickness, (**b**) metal oxide concentration, and (**c**) metal concentration variation of heat-treated samples.

Unlike the single-crystal case, where all the elements are ideally homogeneously distributed throughout the simulation box, here, the spatial distribution is different at the beginning. As a result, the oxide scale is also not homogeneous. Although the oxide layer is primarily formed of Al and Cr oxides, they are observed to be in different clusters and non-uniformly distributed. Essentially, oxidation of the Al-Ni-rich dendrites results in the formation of an α-Al_2_O_3_-rich scale, while the oxidation of the Cr-Fe-rich inter-dendritic region results in the formation of a Cr_2_O_3_-rich scale. Some amount of iron oxide is formed in the Cr-Fe-rich interdendritic regions, while a small amount of NiO is observed in the initial stages in the Al-Ni-rich region. Co is distributed in both of the phases, and a very small amount of CoO forms randomly throughout the scale in the initial stages of oxidation. After 25 h of oxidation, we do not see very clear segregation, as the elements have diffused enough through the dendrite-core–interdendritic interface. Our model does not show significant differences in oxidation kinetics (Figure 7) compared to a single-crystal case. This is understandable since the model did not show a marked difference in α-Al_2_O_3_ vs. Cr_2_O_3_ concentration in the oxide scale for the equiatomic single-crystal simulations. The concentration of Al and Cr oxides is more linear in the heat-treated sample. A similar analysis was also performed for heat treatment at 1473 K, where a larger grain size was observed in the experiments. Figure 8 indicates lower oxide thickness for the 1373 K heat-treated specimen compared to the 1473 K heat-treated one after 25 h of oxidation. As the grain size increases, the kinetics becomes slow due to the reduction of the grain-boundary area (Figure 9). Even though the oxide scale is thicker at 1473 K, the oxide-scale composition does not change appreciably (Figure 10). Although the effect of the grain boundary is not explicitly incorporated into the model, it is still capable of capturing such intricate effect details, thus emphasizing the effectiveness of CA in material modeling. We observed a similar trend in our prior work on Ni-Al and Hf-doped Ni-Al [36].

#### 3.2.2. Precipitate–Matrix Model

Manzoni et al. [42] reported the presence of Cr-Fe-rich precipitates in the dendritic core and interdendritic regions of the AlCoCrFeNi alloy. The dimensions of these precipitates are of the nanoscale order. Being a mesoscale model, CA can handle different dimensions, as the length of the simulation box is virtually appended through scaling. We did not simulate such small dimensions, as the assumptions undertaken in our model are better suited for continuum-level simulations. However, CA is capable of producing meaningful results in a non-dimensional domain. The length of the simulation box is represented in terms of the number of cells and not length units. Likewise, time is also not scaled, and we provide that as the number of iterations.

**Figure 11:**
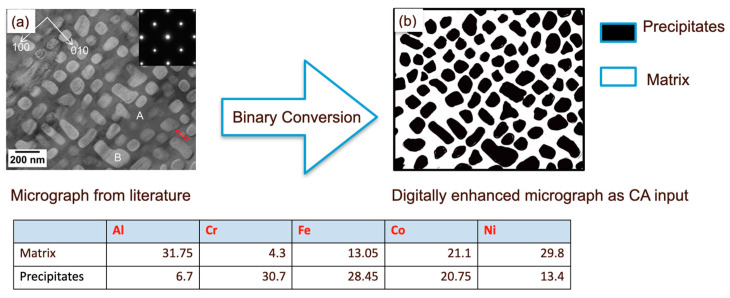
(**a**) Digital micrograph of matrix–precipitate distribution of dendrites in AlCoCrFeNi alloy, as obtained from Reference [44] and converted into (**b**) a binary image, with compositions indicated in the table.

As the compositions differ between Cr-Fe-rich precipitates and the Al-Ni-rich matrix (Figure 11), 110 × 100 matrices are adopted, as opposed to 120 × 50 in the previous cases. The length-to-width ratio also plays an important role in determining the matrix dimension.

**Figure 12:**
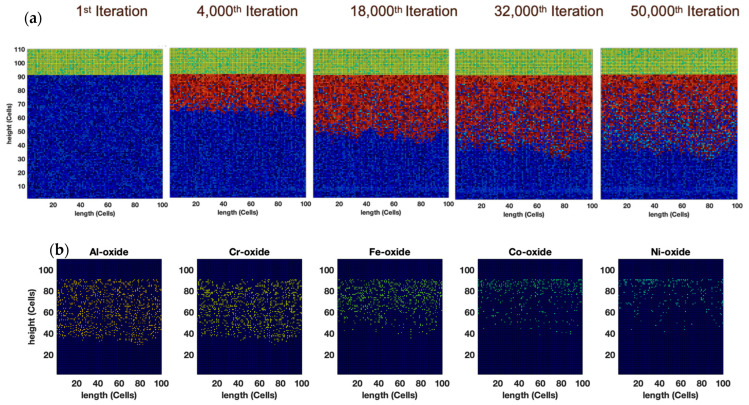
(**a**) Morphology of oxide growth and (**b**) elemental oxide distribution after 50,000 iterations for dendritic matrix–precipitate structure of AlCoCrFeNi alloy. The elemental concentrations were provided in Figure 11.

**Figure 13:**
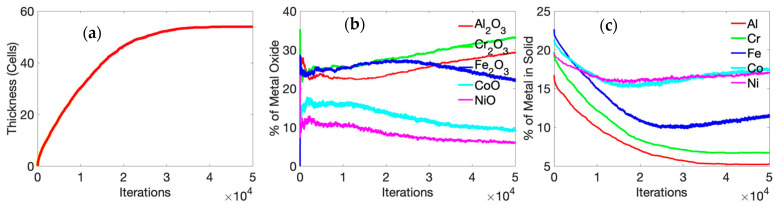
Time-dependent (**a**) thickness, (**b**) metal oxide concentration, and (**c**) metal concentration of dendritic matrix–precipitate structure of AlCoCrFeNi alloy.

Unlike all the other previous cases, the Fe oxide concentration is the highest for oxides between 5000 to 25,000 iterations (Figure 12). As the Fe-Cr-rich precipitate covers more area than the matrix, the overall concentration of Fe in the base alloy is the highest (22 at.%), while Al and Cr concentrations are slightly low, at ~17 and ~19 at.%, respectively (Figure 13). Similar observations were noted for the Al_20_Cr_20_Fe_30_Co_10_Ni_20_ and Al_20_Cr_20_Fe_30_Co_20_Ni_10_ alloys examined earlier.

## 4. Conclusions

We used Cellular Automata to simulate the oxidation behavior of an AlCoCrFeNi high-entropy alloy for a given set of rules. The simulation results match the experimental findings reported in the literature to a reasonable extent, suggesting that we have indeed captured a significant amount of the underlying physics of the process. The key findings may be summarized as follows:

(a) The primary goal of the single-crystal equimolar simulations was to isolate and understand the effect of alloy chemistry. Experimental alloys are, of course, more complex—but the approach adopted is to understand the effect of composition in isolation and then progress toward modeling of multi-phase microstructures. For the equimolar alloy, Al and Cr form the passivating oxides, and the concentration of α-Al_2_O_3_ and Cr_2_O_3_ grows over time in the oxide scale before approaching a steady state. The other oxides (namely those of Fe, Co, and Ni) form initially in larger concentrations, but their concentration in the oxide scale decreases with time. The concentration of CoO and NiO decreases more rapidly than that of iron oxide, possibly due to the greater thermodynamic stability of iron oxide. These results do show a qualitative agreement with experimental findings.
(b) Upon varying the Al:Cr ratio, the overall oxidation kinetics does not change appreciably at 1000 K, but the scale chemistry does change proportional to the Al:Cr ratio in the alloy. Similarly, changing the Fe:Co:Ni ratio did not affect the kinetics significantly. The amounts of oxides of Fe, Co, and Ni in the scale were in accordance with their composition in the alloy. However, a lower Fe content did promote the α-Al_2_O_3_ and Cr_2_O_3_ content in the scale.
(c) The single-crystal simulations, though successfully indicating α-Al_2_O_3_ and Cr_2_O_3_ to be the primary scale-forming oxides, did not indicate a segregation of these oxides; rather, they were seen to form intimately inter-mixed. Once the simulation cell was adjusted to account for the phase-separation observed in the heat-treated samples on which oxidation experiments were performed, we observed a concomitant separation between the Al_2_O_3_- and Cr_2_O_3_-rich scales with the Al-Ni- and Cr-Fe-rich regions in the alloy, respectively.
(d) While the dendritic/interdendritic structures are more typical of traditional casting processes, rapid quenching, followed by aging, can result in the initial formation of a metastable solid solution with extended solid solubility which eventually decomposes, resulting in the formation of precipitates within a matrix. Given the nanoscale distribution of these features, the model could not be scaled, and the results were obtained in terms of the number of iterations/number of cells, as opposed to a scaled time/length, due to lack of experimental data on the oxidation of such alloys. A propensity of iron oxide was observed which could possibly indicate that the number of iterations was successful in capturing only the initial stages. However, here, our results are inconclusive, and further work is required to extend this model for nanoscale features.

From this study, we conclude the following: the oxide scale in HEAs is a function of their complex chemistries and microstructures. We addressed the problem piecewise, studying the oxidation of a single crystal HEA without the microstructural complications and defects to understand the role of alloy chemistry. Subsequently, we considered numerous microstructural features, including compositional segregations observed experimentally, to understand the scale growth in different microstructural regions. We have not yet addressed the problem of internal oxidation using CA. Our future efforts will focus on understanding internal oxidation and subsequently integrate the individual simulations for modeling the microstructures of oxidized HEAs.

## Figures and Tables

**Table 1:** Diffusion coefficients (m^2^/s) of the elements through the oxides as obtained from molecular dynamics by Roy et al. [31] and the conversion into probabilities for CA adoption.

Diffusing Element	Through α-Al_2_O_3_		Through Cr_2_O_3_	
	Diffusion Coefficient	Probability	Diffusion Coefficient	Probability
Al	7.94 × 10^−13^	0.00123456	1.78 × 10^−14^	0.00102564
Cr	6.31 × 10^−12^	0.00138888	2.04 × 10^−15^	0.00093545
Co	1.55 × 10^−11^	0.00147058	9.46 × 10^−16^	0.00090702
Fe	9.77 × 10^−12^	0.00142653	1.26 × 10^−15^	0.00091743
Ni	1.55 × 10^−11^	0.00147058	1.58 × 10^−14^	0.00102040
O	1.01 × 10^−12^	0.06250000	8.13 × 10^−16^	0.04510000

**Table 2:** Diffusion coefficient (m^2^/s) for elements in AlCoCrFeNi HEA, as adapted from Reference [45].

Element	1273	1323	1373
Al	1.00 × 10^−14^	1.00 × 10^−14^	1.00 × 10^−14^
Co	3.26 × 10^−16^	1.09 × 10^−15^	1.55 × 10^−15^
Cr	4.57 × 10^−16^	1.38 × 10^−15^	3.16 × 10^−15^
Fe	3.80 × 10^−16^	1.41 × 10^−15^	2.57 × 10^−15^
Ni	1.00 × 10^−16^	1.66 × 10^−16^	4.78 × 10^−16^

## Data Availability

The data will be made available upon reasonable request to the corresponding author. The code is protected for further development in the field.
